# Molecular Evidence Reveals Taxonomic Uncertainties and Cryptic Diversity in the Neotropical Catfish of the Genus *Pimelodus* (Siluriformes: Pimelodidae)

**DOI:** 10.3390/biology13030162

**Published:** 2024-03-02

**Authors:** Daniel Limeira Filho, Elidy Rayane de Rezende França, Dalton Kaynnan de Prado Costa, Renato Correia Lima, Maria Histelle Sousa do Nascimento, Jacqueline da Silva Batista, Maria Claudene Barros, Elmary da Costa Fraga

**Affiliations:** 1 Graduate Program in Animal Science—PPGCA, Center of Agrarian Sciences—CCA, Maranhão State University—UEMA, São Luís 65055-310, MA, Brazil; elidyany@hotmail.com (E.R.d.R.F.); mbdene@yahoo.com.br (M.C.B.); 2 Graduate Program in Biodiversity, Environment, and Health—PPGBAS, Caxias Center of Higher Education—CESC, Maranhão State University—UEMA, Praça Duque de Caxias, s/n-Morro do Alecrim, Centro, Caxias 65604-380, MA, Brazil; daltonprado322@gmail.com; 3 Graduate Program in Genetics, Conservation, and Evolutionary Biology (PPG-GCBEv), National Amazonian Research Institute–INPA, Av. André Araújo, 2936, Aleixo, Manaus 69060-001, AM, Brazil; rcl.rcl.rcl.rcl.lima@gmail.com (R.C.L.); jacqueline.batista@posgrad.inpa.gov.br (J.d.S.B.); 4 Graduate Program in Biodiversity and Biotechnology—BIONORTE Network, Maranhão State University—UEMA, Cidade Universitária Paulo VI—Avenida Lourenço Vieira da Silva, n° 1.000, Jardim São Cristóvão, São Luís 665055-310, MA, Brazil; histelle.sousadonascimento@gmail.com; 5 Molecular Biology Thematic Laboratory—LTBM, Coordination of Biodiversity—COBIO, National Amazonian Research Institute–INPA, Av. André Araújo, 2936, Petrópolis, Manaus 69067-375, AM, Brazil; 6 Laboratory of Molecular Biology—LABMOL, Department of Chemistry and Biology, Caxias Center of Higher Education—CESC, Maranhão State University—UEMA, Praça Duque de Caxias, s/n-Morro do Alecrim, Centro, Caxias 65604-380, MA, Brazil; 7 Laboratory of Genetics—LABGEN, Department of Chemistry and Biology, Caxias Center of Higher Education—CESC, Maranhão State University—UEMA, Praça Duque de Caxias, s/n-Morro do Alecrim, Centro, Caxias 65604-380, MA, Brazil

**Keywords:** neotropical region, cryptic species, freshwater fish, catfish, Cytochrome *c* oxidase subunit I

## Abstract

**Simple Summary:**

The catfish of the genus *Pimelodus* are amply distributed in the Neotropical region, although the species-level taxonomy and phylogenetic relationships of these fish are still poorly resolved. In the present study, we used a molecular approach to delimit the *Pimelodus* species from the different river basins of the Neotropical region. For this, we analyzed sequences of the mitochondrial Cytochrome *c* oxidase subunit I (COI) gene from 13 nominal species, which generated 24 consensus Molecular Operational Taxonomic Units (MOTUs). Only six of the nominal species were recovered as well-defined molecular entities, while seven presented cryptic diversity or taxonomic uncertainties. The DNA barcode analysis presented here represents an important step toward the definition of the species of this economically important group of fish, which will be fundamental to the conservation of its diversity.

**Abstract:**

*Pimelodus* is the most speciose genus of the family Pimelodidae, and is amply distributed in the Neotropical region. The species-level taxonomy and phylogenetic relationships within this genus are still poorly resolved, however. These taxonomic problems and the general lack of data have generated major uncertainties with regard to the identification of specimens from different localities. In the present study, we applied a single-locus species delimitation approach to identify the MOTUs found within the genus *Pimelodus* and provide sound evidence for the evaluation of the species richness of this genus in the different river basins of the Neotropical region. The study was based on the analysis of sequences of the mitochondrial COI gene of 13 nominal species, which resulted in the identification of 24 consensus MOTUs. Only six nominal species were recovered as well-defined molecular entities by both the traditional barcoding analysis and the molecular delimitation methods, while the other seven presented cryptic diversity or persistent taxonomic uncertainties. The lineages identified from the Parnaíba ecoregions, Amazonas Estuary and Coastal Drainages may represent a much greater diversity of *Pimelodus* species than that recognized currently, although a more detailed study of this diversity will be necessary to provide a more definitive classification of the genus.

## 1. Introduction

The catfish family Pimelodidae is endemic to the Neotropical region, and is one of the most diverse families of the order Siluriformes, with 30 genera and 116 valid species [1]. Many of these species are important resources for both subsistence and commercial fisheries in South America [2]. The pimelodids have a typical catfish morphology, with body coloration ranging from a uniform gray to elaborate patterns of stripes and spots [3].

The fish of the genus *Pimelodus* LaCépède, 1803, the most speciose of the family Pimelodidae, are distributed between Panama and the River Plate in northern Argentina [4,5,6]. While a total of 36 valid *Pimelodus* species are currently recognized, the systematics of this genus are considered to be among the most problematic of any pimelodid taxon [7,8]. As no apomorphic trait has been accepted universally for the genus, the reliable identification of species remains a challenge [9]. In fact, the diagnosis of the genus *Pimelodus* has long been based on a set of phylogenetically non-informative traits, which are inadequate for the definition of a monophyletic taxon [10].

Overall, then, the large number of species currently included in the genus *Pimelodus*, together with the variability found in the morphology and coloration patterns of the different forms, has hampered a more definitive review of the systematics of the genus [11,12]. This means that not only the species-level taxonomy of the genus but also its phylogenetic relationships are still poorly understood [7,11,12]. A number of phylogenetic studies have demonstrated the complexity of this taxon, which still has unresolved questions on the status of species and their monophyly. In addition, the species with a more ample distribution, such as *Pimelodus blochii* Valenciennes, 1840, *Pimelodus pictus* Steindachner, 1876, and *Pimelodus ornatus* Kner, 1858, appear to represent species complexes [7,8,13].

Many of the valid *Pimelodus* species are still poorly known and, given their morphological complexity, they may, in fact, represent multi-species lineages that have yet to be identified systematically. In this context, the use of molecular data will be essential to evaluate patterns of inter-basin differentiation [7] and delimit species, in particular in the cases in which the morphology-based identification is inadequate, thus revealing diversity that has been underestimated or not yet recognized scientifically [14,15,16,17]. Padial et al. [18] concluded that the current systematics advanced with the progressive inclusion of molecular studies, which has pointed increasingly to the existence of distinct units that may represent candidate species.

While morphological traits are fundamental to the identification of most organisms, ongoing technological advances have led to the increasing use of alternative criteria to define species [19]. In recent years, a growing number of publications on the species concept, and taxon-delimitation methods and their applications, have revived taxonomic research [20]. In 2003, a standardized system for the molecular identification of species was proposed based on a specific segment of the DNA sequence of a mitochondrial gene, an approach known as DNA barcoding [21]. More recent studies have adopted a number of alternative procedures for the molecular delimitation of species, using analytical approaches that involve different methods for the identification of Molecular Operational Taxonomic Units, or MOTUs [14,16,17,22].

A recent study of *Pimelodus*, based on the use of molecular markers, confirmed the existence of two independent evolutionary lineages from the trans-Andean region, which were described as new species [23]. Studies of this type are still limited in the Brazilian Northeast, however, and most of the *Pimelodus* taxa known to occur in the hydrographic basins of the state of Maranhão have been catalogued based solely on classical taxonomic criteria [24]. In addition, given the confused taxonomy of the genus, the nomen *P. blochii*, for example, has been attributed arbitrarily to many types of long-whiskered catfish from different localities in the Amazon basin, which likely represent more than one species [25]. A similar situation likely applies to the basins of Maranhão, given that some specimens from this region have also been identified as *P. blochii*. In this context, the integration of complementary studies that employ molecular tools will be essential to ensure the more reliable diagnosis of species and a better understanding of the diversity of this genus.

The present study employed a number of single-locus species delimitation approaches to identify the MOTUs present within the genus *Pimelodus*, focusing on the hydrographic basins of the Brazilian state of Maranhão, and the compilation of evidence for a more conclusive diagnosis of the species richness of this genus. For this, samples of specimens from a number of different localities across the Neotropical region were included in the analyses. This makes the present study the most comprehensive yet to diagnose the limits of the valid species that make up the complex diversity of this genus.

## 2. Materials and Methods

### 2.1. Sampling

**Figure 1:**
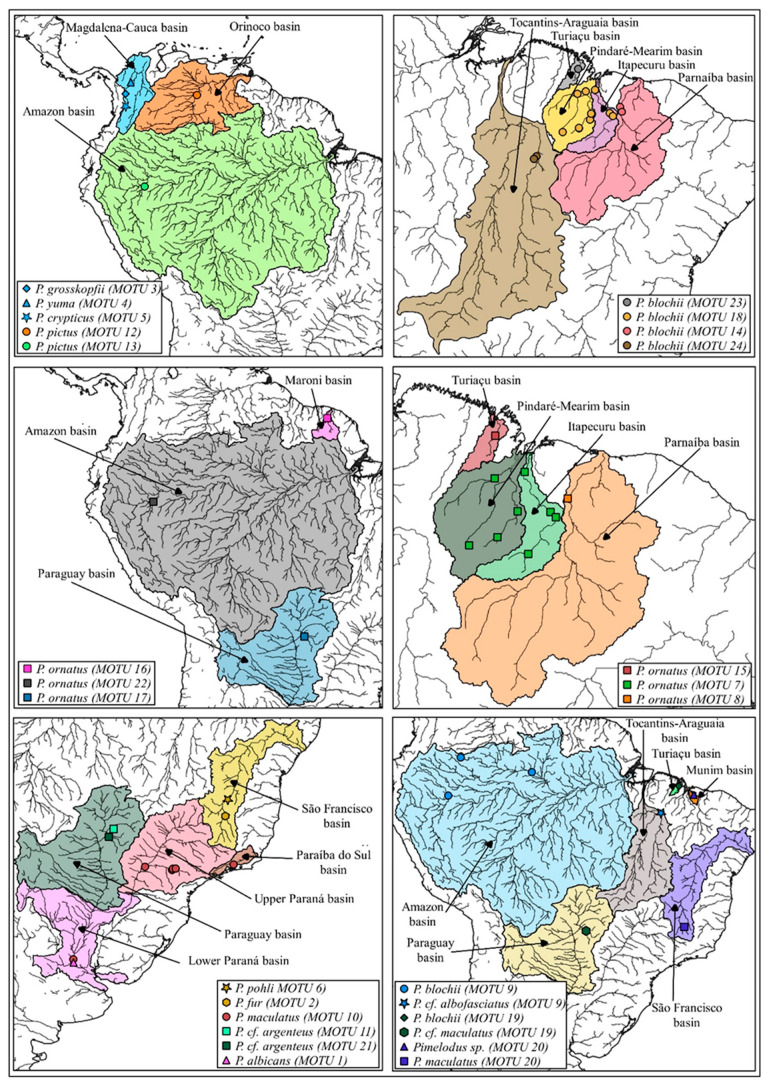
Collecting localities (symbols) and the hydrographic basins of the Neotropical region in which the MOTUs of the genus *Pimelodus* were identified in the present study.

In the present study, a total of 257 specimens representing 13 *Pimelodus* species were obtained from a number of different hydrographic basins distributed across South America (Figure 1 and Supplementary Tables S1 and S2). The majority (174) of these specimens were collected during the present study, between 2014 and 2022, in the basins of the Itapecuru, Mearim, Pindaré, Turiaçu, Munim, Parnaíba, and Tocantins rivers, in the Brazilian states of Maranhão, Piauí, and Tocantins (Supplementary Table S1). Samples of muscle tissue were preserved in 96% ethanol and then refrigerated at −20 °C prior to the implementation of the molecular procedures.

**Figure 2:**
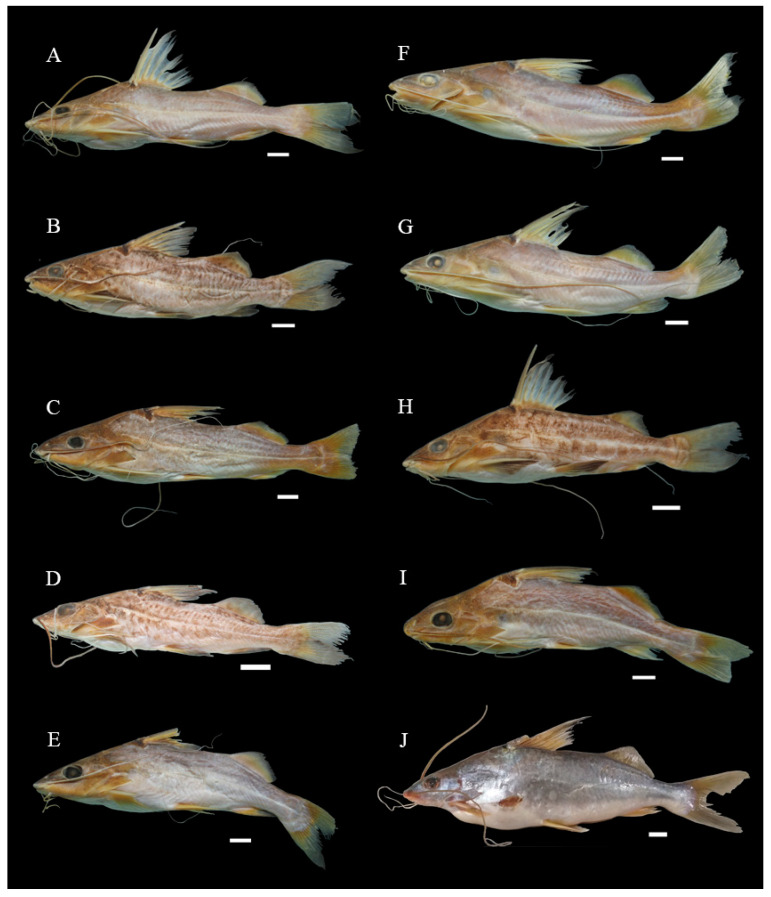
Specimens of *Pimelodus blochii* analyzed in the present study (and the river from which each specimen was collected): (**A**) *P. blochii* (Mearim); (**B**) *P. blochii* (Pindaré); (**C**) *P. blochii* (Grajaú); (**D**) *P. blochii* (Flores); (**E**) *P. blochii* (Corda); (**F**) *P. blochii* (Itapecuru); (**G**) *P. blochii* (Parnaíba); (**H**) *P. blochii* I (Turiaçu); (**I**) *P. blochii* II (Turiaçu), and (**J**) *P. blochii* (Tocantins). Scale bar = 1 cm. Photographs: Daniel Limeira, Renato Correia.

**Figure 3:**
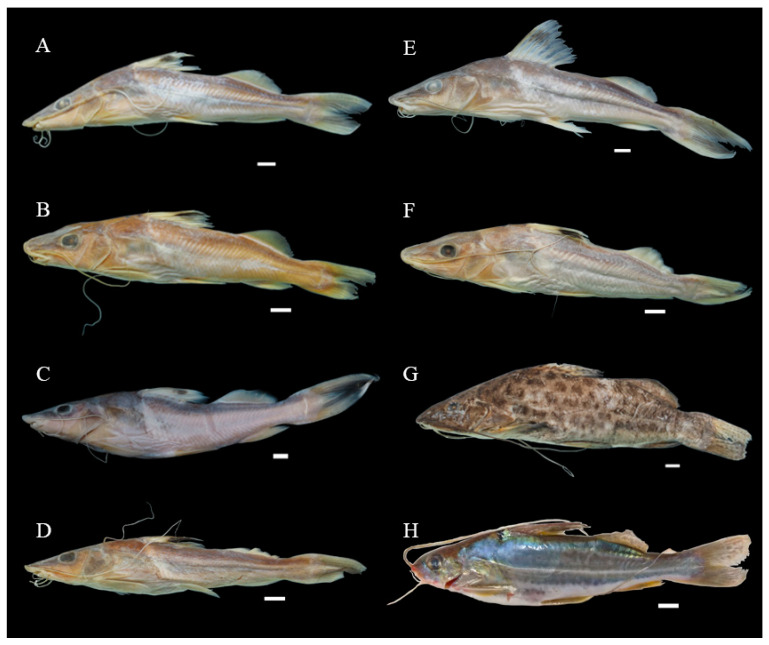
Specimens of *Pimelodus* analyzed in the present study (and the river from which each specimen was collected): (**A**) *P. ornatus* (Itapecuru); (**B**) *P. ornatus* (Mearim); (**C**) *P. ornatus* (Pindaré); (**D**) *P. ornatus* (Grajaú); (**E**) *P. ornatus* (Parnaíba); (**F**) *P. ornatus* (Turiaçu); (**G**) *Pimelodus* sp. (Munim), and (**H**) *P.* cf. *albofasciatus* (Tocantins). Scale bar = 1 cm. Photographs: Daniel Limeira, Renato Correia.

All the recognized nominal species were represented in the sample by at least two individuals. Voucher specimens were deposited in three established Brazilian ichthyological collections, the Fish Zoological Collection of the National Amazonian Research Institute (INPA) in Manaus, and the collections of the Museum of Zoology of the University of São Paulo (MZUSP) in São Paulo and the Museum of Zoology of Londrina State University (MZUEL) in Londrina. The remaining specimens are held in the Molecular Biology Laboratory (LABMOL) of the GENBIMOL complex of the Caxias campus of Maranhão State University (UEMA) in Brazil. Photographic vouchers were also used whenever it was not possible to deposit a physical specimen in an ichthyological collection (Figure 2 and Figure 3 and Supplementary Table S1).

The database analyzed in the present study included 83 COI sequences of *Pimelodus* specimens collected during previous studies in a number of different South American basins (Supplementary Table S2). These areas include the Maroni River [26], the basin of the Magdalena–Cauca and Orinoco Rivers [23], the Amazon River [27,28], the São Francisco River [29,30], the Paraíba do Sul River [31], the Paraguay River [32], the upper and lower Paraná basins [33,34,35,36], and the plains of the Argentinian Pampa [37]. In all these cases, the taxonomic identification of the species followed the original denomination presented in the respective study.

### 2.2. Extraction, Amplification, and Sequencing of the DNA

The total DNA was extracted from the muscle tissue using Promega’s Wizard Genomic DNA Purification kit, which was implemented following the maker’s recommendations. The target region of the Cytochrome *c* Oxidase Subunit I (COI) gene was amplified by Polymerase Chain Reaction (PCR) using universal primers [38].

The PCRs were run in a final volume of 25 μL, which contained 2 µL of the DNA (250 ng/ µL), 4 µL of nucleotide dNTPs (1.25 M), 2.5 µL of 10x buffer solution, 0.5 µL of MgCl_2_ solution (50 mM), 0.25 µL of each primer (200 ng/ µL), 0.2 µL of Taq polymerase (5 U/ µL), and purified water to complete the final reaction volume. The samples were amplified using the following cycle: initial denaturation at 95 °C for 2 min, followed by 35 cycles at 94 °C for 30 s (denaturation), 54 °C for 30 s (annealing), and 72 °C for 1 min (extension), with a final extension of 10 min at 72 °C.

The PCR products were purified using the ExoSap IT kit and the DNA was sequenced using the Sanger et al. [39] method. The precipitated products were sequenced in an ABI Prism™ 3500 automatic DNA sequencer (Applied Biosystems, Waltham, MA, USA).

### 2.3. The DNA Barcoding Analysis and Delimitation of the MOTUs

The COI sequences obtained by the PCR were edited manually in BIOEDIT 7.0 [40] and aligned in CLUSTAL W 1.4 [41]. All the sequences generated in the present study were deposited in GenBank (https://www.ncbi.nlm.nih.gov/genbank/ accessed on 16 January 2024) under the following accession numbers: “PP132870–PP133043”(Table S1). The translation of the nucleotide sequences into amino acids did not reveal any indels or stop codons. The *Pimelodus* species were delimited through the application of five different approaches to the COI dataset: (i) Automatic Barcode Gap Discovery—ABGD [42], (ii) Assemble Species by Automatic Partitioning—ASAP [43], (iii) Multi-rate Poisson Tree Processes—mPTP [44], (iv) the Bayesian implementation of the PTP model—bPTP [45], and (v) the General Mixed Yule Coalescent—GMYC [46].

The SPdel pipeline (species delimitation), a tool developed recently to visualize and compare single-locus species delimitation methods, was also employed here. This tool was designed to calculate indices and compare the MOTUs obtained by the different methods, such as the ASAP, GMYC, bPTP, mPTP, and BIN approaches used here. One of the principal functions of the SPdel pipeline is to compare the different delimitation methods and generate consensus MOTUs [47]. The SPdel estimates intraspecific and interspecific genetic distances, based on the Kimura 2-parameter (K2P) model [48]. In this case, the mean intra-MOTU and the maximum intra-MOTU distances were calculated, as well as the Nearest Neighbor (NN) and the minimum distance to the NN, for both the nominal species and the consensus MOTUs.

The dataset of the COI sequences aligned in the Fasta format was used as the input file for the ABGD and ASAP methods, while the input for the mPTP, bPTP, and GMYC models was an ultrametric Bayesian (BI) tree, generated in BEAST v.1.10.4 [49,50], which was run on the CIPRES Science Gateway web server [51]. This analysis was run using a strict molecular clock and Yule model prior to the speciation process. The phylogenetic tree was inferred using the TN93 substitution model [52] with Gamma distribution, which was selected using jModelTest v.2.1.10 [53], with the application of the Bayesian Information Criterion (BIC). A single run of 100 million Markov Chain Monte Carlo (MCMC) generations was implemented, with the initial 10% of the runs being discarded as burn-in. The convergence and Effective Sample Size (ESS > 200) were evaluated using Tracer v. 1.7 [54]. The dataset was used subsequently to generate a consensus tree in Treeannotator v. 1.10.4 [50]. The phylogenetic tree obtained from these analyses was visualized and edited using Fig Tree v1.4.4 [55] and the Inkscape v1.1 image-editing system [56].

The DNA barcoding approach [21] followed monophyly-based criteria (tree-based method) and the analysis of genetic distances was based on a predefined threshold of molecular divergence as the cutoff point for the delimitation of species. In Neotropical fish, this value is typically 2% [33], but rather than using this standard value, an Optimized Threshold (OT) was calculated here, based on the dataset generated in the present study. For this, the LocalMinima function was run in the SPIDER package [57] of R, version 4.3.1 [58].

In addition to the SPdel tool, MEGA X [59] was used to calculate a pairwise K2P distance matrix to compare the maximum intraspecific and minimum interspecific distances for each nominal species, as well as a matrix of the mean inter-MOTU genetic distances (Supplementary Table S3). The taxonomic units were plotted on a quadrant graph using the threshold calculated in the present study as the reference value [60]. The nomenclature of Hebert et al. [60], which was adapted from Machado et al. [14], was used to denominate the distribution of these units among the different quadrants. A Neighbour-Joining (NJ) tree [61] was generated using the K2P model to represent visually the divergences among the study species (Supplementary Figure S1). The significance of the groups formed in this analysis was estimated by a bootstrap analysis, with 1000 pseudo-replicates [62].

## 3. Results

**Figure 4:**
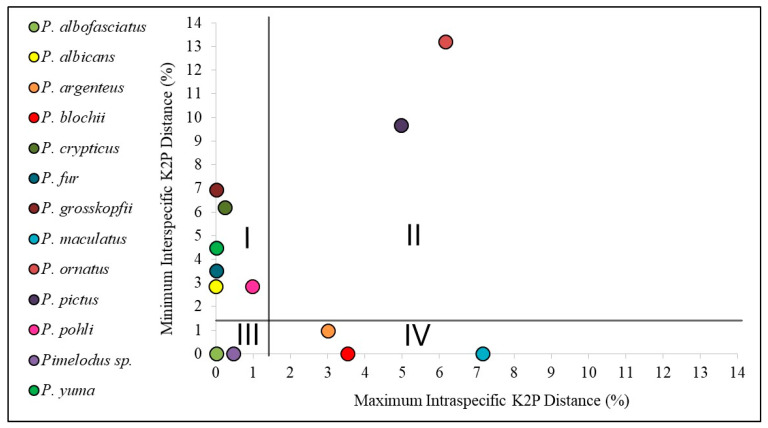
Comparison of the intra- and interspecific COI distances among the nominal *Pimelodus* species, allocated to the four quadrants of the plot, which represent: (I) total agreement between the molecular and morphological identifications; (II) the possible existence of cryptic species; (III) recent divergence, synonymy, or hybridization; and (IV) lack of correspondence between the taxonomy and the molecular identification.

The final alignment of the mitochondrial COI dataset provided 257 sequences (174 generated here and 83 from GenBank), a majority of which had 643 base pairs (bps), with some sequences having fewer bps, down to a minimum of only 442 bps. The SPIDER LocalMinima function indicated a species threshold of 0.0148 (that is, a divergence of 1.48%). The taxonomic units defined here were distributed in all four quadrants relative to this threshold (Figure 4).

Quadrant I includes *Pimelodus albicans* (Valenciennes, 1840), *Pimelodus crypticus* Villa-Navarro & Cala, 2017, *Pimelodus fur* (Lütken, 1874), *Pimelodus grosskopfii* Steindachner, 1879, *Pimelodus pohli* Ribeiro & Lucena 2006, and *Pimelodus yuma* Villa-Navarro & Acero P. 2017. In all these cases, the intraspecific distances were lower than 1.48%, while the interspecific distances were all above this threshold, with complete agreement between the morphological and molecular identifications.

Quadrant II contains *P. ornatus* and *P. pictus*, with both intraspecific and interspecific distances of over 1.48%, which indicates the possible existence of cryptic species (candidates for taxonomic division). By contrast, the two species in quadrant III—*Pimelodus albofasciatus* Mees, 1974 and *Pimelodus* sp.—both returned intraspecific and interspecific distances lower than 1.48%, which indicate recent divergence, synonymy, hybridization, or errors of identification.

The three remaining species, *Pimelodus argenteus* Perugia 1891, *Pimelodus maculatus* LaCépède, 1803, and *P. blochii*, were assigned to quadrant IV, in which the intraspecific distances were over 1.48%, while the interspecific distances were lower than this. This scenario reflects a lack of correspondence between the morphological and molecular identifications.

**Figure 5:**
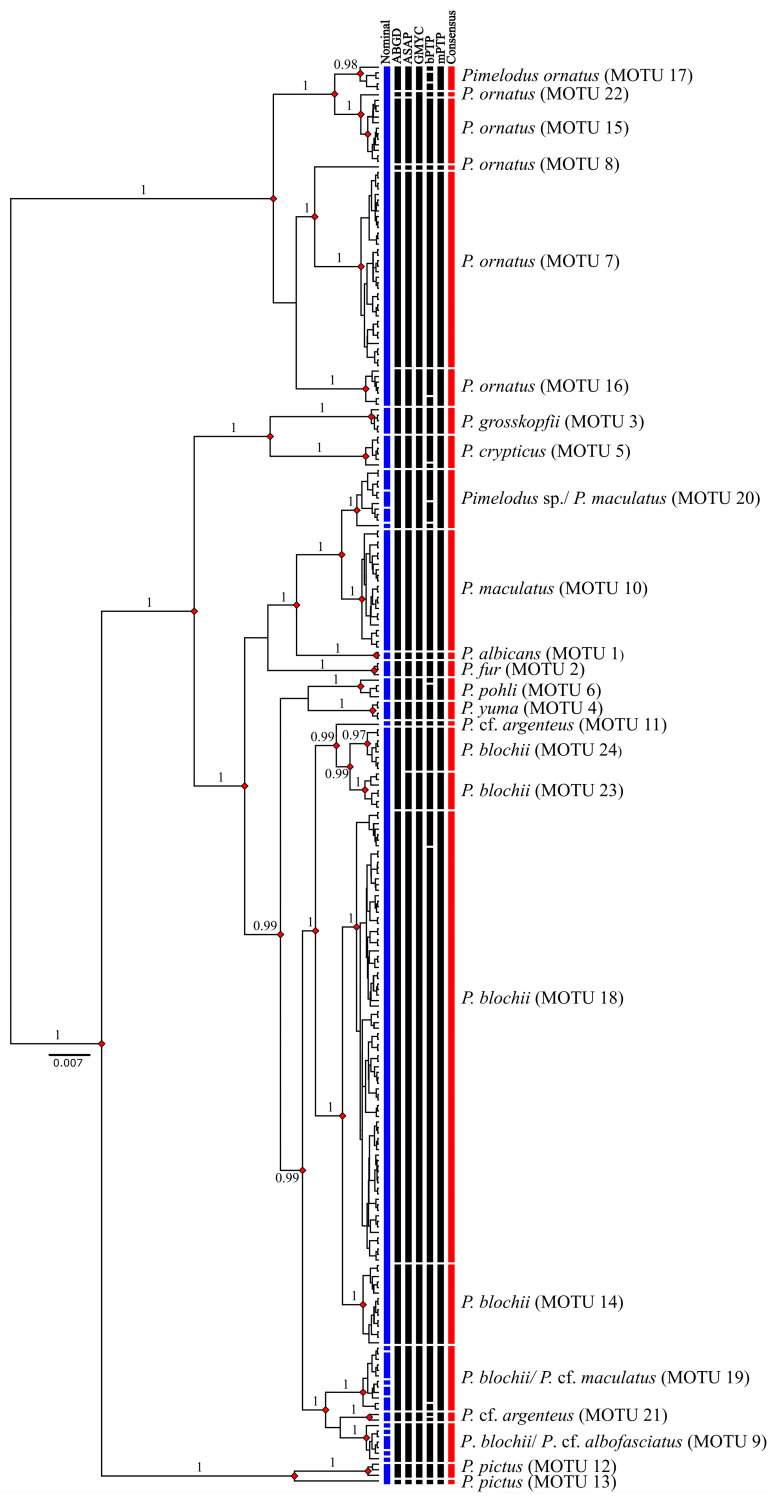
Bayesian Inference (BI) tree, showing the arrangement of the *Pimelodus* MOTUs obtained by the species delimitation analyses. The blue line represents the nominal taxonomy and the red line represents the consensus MOTUs. The red diamonds indicated the nodes with a Bayesian posterior probability of over 0.96 (values shown on the branches).

The molecular delimitation of the species, by both the ABGD (adopting a recursive partition based on the *p* values closest to 0.01) and GMYC methods (which presented a significant likelihood ratio test, with *p* < 0.0001) identified 23 MOTUs, whereas the ASAP (based on the partition with the lowest ASAP score) and mPTP indicated 24 MOTUs (Figure 5). The bPTP analysis defined 34 MOTUs, a much higher number of molecular entities in comparison with the preceding methods (Figure 5). The SPdel pipeline compiled the results of the different delimitation methods to reveal a consensus of 24 MOTUs (Figure 5), with a mean of 10.7 individuals per MOTU, ranging from 1 to 82 individuals. Four MOTUs were represented by a single specimen. The groups formed in the phylogenetic analyses were congruent and well-supported with significant posterior probability (BI) and bootstrap (NJ) values overall (Figure 5; Supplementary Figure S1).

Four of the twenty-four consensus MOTUs, which correspond to four of the nominal species, i.e., *P. albicans*, *P. fur*, *P. grosskopfii*, and *P. yuma*, were recovered in all the analyses applied here (Figure 5). Two MOTUs, representing *P. crypticus* and *P. pohli*, were also obtained by all the methods, except for the bPTP delimitation (Figure 5).

A clear division was observed in *P. ornatus*, with six MOTUs representing the populations from the Amazon, Maroni, Paraguay, Turiaçu, Itapecuru, Mearim-Pindaré, and Parnaíba basins, while *P. argenteus* from the Paraguay basin and *P. pictus* from the Orinoco and Amazon basins each formed two MOTUs. All of these arrangements were supported by four or more of the species delimitation methods employed in the present study (Figure 1 and Figure 5).

Four MOTUs were identified in *P. blochii*, based on specimens from the Tocantins-Araguaia, Turiaçu, Itapecuru, Mearim-Pindaré, and Parnaíba basins, as two MOTUs that were shared with other nominal species (Figure 1). A similar situation was observed in *P. maculatus*, which formed a MOTU made up of specimens from the Paraíba do Sul and upper and lower Paraná basins, with two other MOTUs also being shared (Figure 1).

Three MOTUs were formed by the conjunction of species considered to be distinct and geographically allopatric. The first of these MOTUs was composed of *P. blochii* from the Amazon basin, together with *P.* cf. *albofasciatus* from the Tocantins basin (Figure 1), while the second included a group of *P. blochii* specimens from the Turiaçu basin, together with *P.* cf. *maculatus* from the basin of the Paraguay River (Figure 1). The third MOTU was made up of *Pimelodus* sp. from the Munim basin, together with *P. maculatus* from the basin of the São Francisco River (Figure 1). It is interesting to note that the *P. blochii* specimens from the Turiaçu basin were divided into two distinct MOTUs, one of which is described above (Figure 1). All of these arrangements are supported by at least four of the species delimitation analyses applied here (Figure 5).

The mean intra- and maximum intra-MOTU distances, the Nearest Neighbor (NN), and the minimum distance to the NN of both the nominal species and the consensus MOTUs are shown in Table 1. The maximum intra-MOTU distance was 0.98%, which was recorded within both *P. pohli* (MOTU 6) and *P. maculatus* (MOTU 10), while the minimum inter-MOTU distance was 0.78% between the *P. blochii* MOTUs. In the case of the nominal species, the maximum intraspecific distance was 7.15% (within *P. maculatus*), while minimal distances of zero were recorded between individuals of *P. albofasciatus*, *P. blochii*, *P. maculatus*, and *Pimelodus* sp. (Table 1). Considering the genetic divergence threshold of 1.48% (maximum intra- vs. minimum inter-), only 13 MOTUs would be located within quadrant I, while all the others would be in quadrant III, with minimum inter-MOTU distances of 0.78–1.18% (Table 1). The comparisons of the mean intra- and inter-MOTU genetic distances are shown in Supplementary Table S3.

## 4. Discussion

Given the challenges of determining biological diversity accurately and reliably, the identification of MOTUs provides a rapid diagnosis of diversity and the occurrence of potential candidate species. As a reference, Ramirez et al. [17] found evidence of cryptic diversity in the fish genus *Schizodon* Agassiz, 1829, and this evidence supported a new study in which a new *Schizodon* species was described from the basins of the Xingu and Tapajós rivers [63].

Similar results were obtained from the molecular delimitation of the genus *Salminus* Agassiz, 1829 [14] and the recently described *Megaleporinus* Ramirez, Birindelli & Galetti, 2017 [16], with additional biological units being observed in both cases. In the most recent taxonomic review of the small-bodied dorados, fish of the genus *Salminus*, the findings of Machado et al. [14,64] were corroborated partially, culminating in the description of a new species from the Tocantins–Araguaia basin [65]. Given these findings, it would seem likely that at least some of the nominal *Pimelodus* species included in the present analysis contain cryptic diversity and as yet undescribed taxa. In fact, in both the traditional barcoding and molecular delimitation applied here, only six of the nominal species (*P. albicans*, *P. crypticus*, *P. fur*, *P. grosskopfii*, *P. pohli*, and *P. yuma*) were recovered as well-defined molecular entities, while all the other seven (*P. albofasciatus*, *P. argenteus*, *P. blochii*, *Pimelodus* sp., *P. maculatus*, *P. ornatus*, and *P. pictus*) presented some level of taxonomic uncertainty.

The DNA barcoding and species delimitation analyses indicated the existence of cryptic species, recently diverged taxa, synonyms, and possible cases of erroneous identification in the 13 taxa that were delimited into a total of 24 consensus MOTUs. Previous molecular delimitation studies of a range of different fish groups have all found a greater number of molecular entities, in comparison with the recognized species richness [14,15,16,17,22,66,67].

The relatively large number of molecular entities detected in the present study reflects the low genetic distance values (0.78–0.79%) recorded between MOTUs within the genus *Pimelodus*, most of which are arranged in allopatric lineages (Figure 1, Table 1): *P. blochii* from the Parnaíba basin (MOTU 14), *P. blochii* from the Itapecuru and Pindaré–Mearim basins (MOTU 18), a group of *P. blochii* individuals from the Turiaçu basin (MOTU 23), and *P. blochii* from the Tocantins basin (MOTU 24) as well as *P. ornatus* from the Turiaçu (MOTU 15) and *P. ornatus* from the Amazon basin (MOTU 22). It seems likely that these reduced genetic distances reflect the recent divergence or may represent distinct genetic populations.

As Ramirez et al. [17] point out, scenarios of this type may arise principally when a barcoding analysis focuses on a group of intimately related species, such as those of the same genus. Similar situations have been observed in a number of studies of fish genera, such as *Astyanax* Baird & Girard 1854 [67], *Megaleporinus* [16], *Laemolyta* Cope, 1872 [68], *Leporinus* Agassiz, 1829 [15,69], *Prochilodus* Agassiz, 1829 [70], and *Brycon* Müller & Troschel 1844 [71]. Even so, an integrated approach including multiple traits would be necessary to best determine whether the MOTUs found in the present study actually represent recent speciation or simply a high level of population structuring.

The nominal species *P. ornatus* was divided among six MOTUs, for example, with minimum inter-MOTU distances of 0.79–2.91% (Figure 1, Table 1). Four of these six MOTUs were separated by genetic distances of at least 1.48% (Table 1), including those that represent populations which inhabit the hydrographic basins of Maranhão, supporting the hypothesis of cryptic diversity. Evidence of potential cryptic speciation was also found in *P. pictus*, which formed two MOTUs, separating the genetic lineages of the Orinoco and Amazon basins (Figure 1, Table 1).

These findings reinforce the conclusion that these taxa are candidates for taxonomic division, which further supports Lundberg et al. [7], who showed that individuals from the allopatric populations of nominal species such as *P. ornatus* and *P. pictus* presented genetic divergence as least as accentuated as that of different species of the same genus. In addition, the lack of a clear evolutionary relationship between some of the species included in the genus *Pimelodus* (e.g., *P. ornatus*) supports the reallocation of these taxa to a different genus or even a new, as yet undescribed genus [8,10].

Three individuals of the nominal species *P. argenteus* from the Paraguay basin included in the present analysis formed two MOTUs (Figure 1, Table 1) separated by high levels of genetic divergence (Supplementary Table S3), as found by Lima et al. [32], who denominated the lineages clades A and B. These authors applied a 2% genetic divergence cutoff point, which indicates possible sub-structuring in this population that could only be confirmed by a more detailed study in population genetics. The 2% divergence threshold as a heuristic cutoff value for species delimitation is considered a good starting point for molecular identification of ichthyofauna [29,31,33]. However, values lower than 2% have already been reported in the literature to indicate candidate species [14] and even to separate congeneric species [63].

The novel finding obtained here refers to the strict relationship that exists between one *P.* cf. *argenteus* specimen (MOTU 11), which corresponds to clade B, *sensu* Lima et al. [32], and *P. blochii* (MOTU 24) from the Tocantins–Araguaia basin (minimum inter-MOTU distance = 0.81%). The latter MOTU (24) also presents an inter-MOTU distance of 0.78% from the *P. blochii* (MOTU 23) of the Turiaçu basin (Table 1). These intimately related MOTUs were separated by the criterion of monophyly and their allopatric distribution (Figure 1, 5), which indicates a possibly recent speciation event.

The geographic distribution of *P. argenteus* includes the basins of the lower Paraná River and the Paraguay River [72]. This species is identified by the lack of spots on the body, with coloration ranging from dark chestnut to silvery gray [9], darker on the back and flanks (down to the lateral or slightly lower), and lighter on the venter [73]. The dorsal aculeus is well-developed and reaches the adipose fin when depressed [9]. While a large number of samples are available of *P. blochii* from the Tocantins basin (MOTU 24) and the group of *P. blochii* (MOTU 23) from the Turiaçu basin, *P.* cf. *argenteus* (MOTU 11) was represented by only a single sequence, obtained from a specimen that was not available for analysis, which is why this arrangement was not discussed in more detail here.

In *P. maculatus*, one MOTU included specimens from the Paraíba do Sul and upper/lower Paraná basins, with a greater intra-MOTU distance (Table 1, Figure 1). A number of studies have reported a certain degree of genetic diversity among the populations of *P. maculatus*, but not enough to consider them distinct species [34,74,75]. Curiously, both the nominal *P. maculatus* and *P. blochi* were included in MOTUs that included specimens of the other nominal species analyzed here. In the specific case of *P. maculatus*, one of these arrangements involved *P.* cf. *maculatus* from the Paraguay basin with a group of specimens of *P. blochii* from the basin of the Turiaçu River, in the Brazilian state of Maranhão (Figure 1).

Given the discrepancies observed here, it is important to emphasize that the *P.* cf. *maculatus* specimens from the Paraguay basin included in the present analyses were identified as such in the study of Lima et al. [32]. Comparing the data on the sampling points of the specimens analyzed in the present study with the known distribution of the *Pimelodus* species, it seems likely that the *P.* cf. *maculatus* specimens were identified erroneously by these authors, and that these specimens are in fact members of a species distinct from *Pimelodus maculatus* LaCepède, 1803. This would account for the accentuated genetic divergence of these specimens from the *P. maculatus* MOTUs of the other basins surveyed here (Figure 5, Supplementary Table S3).

Lima et al. [32] based their taxonomic designation of the specimens collected in their study on the manual of Britski et al. [73]. However, Souza-Filho and Shibatta [9] described a new species, *Pimelodus pantaneiro*, from this study area, for example, which was denominated previously as *P. maculatus* by Britski et al. [73,76]. This newly described species is easily confused with *P. maculatus*, in particular, in terms of its coloration, given that both species have three to five large spots on the flanks [9]. These authors also found evidence of the occurrence of four other species from the region of the upper Paraguay River; that is, *P. ornatus*, *P. argenteus*, *P. absconditus*, and *P. mysteriosus*. However, this does not explain why the *P. blochii* group from the Turiaçu basin aligns with the specimens of this species from the Paraguay basin in a single MOTU, given their geographic distribution and the morphological traits that distinguish them.

Two “varieties” of *P. blochi*—*A* and *B*—have been described. While variety *A* has a uniform gray body with no spots or stripes, variety *B* has four dark lateral stripes, of which the fourth may be absent or fragmented into dots [77]. In addition, the minimum inter-MOTU distance observed here was 1.18%, which is consistent with the variation within a nominal species (Table 1), indicating a much more complex scenario. The uncertainties of the status of these entities can only be resolved through more comprehensive and consistent morphological comparisons, together with a more extensive analysis, including additional molecular markers, which are nevertheless beyond the scope of the present study.

In the Turiaçu basin, the molecular differences found within the *P. blochii* population were sufficient to form two distinct MOTUs, one of which was mentioned above, with a mean inter-MOTU distance of 2.88% (Supplementary Table S3). When the MOTUs formed by individuals considered to be members of the same species, but from different basins in eastern Maranhão, were compared, the divergence values were over 2%, which indicates the existence of cryptic diversity in this region (Supplementary Table S3). The morphological characteristics of the specimens collected from the Turiaçu basin are consistent with those of Eigenmann [77] *A* and *B* varieties (Figure 2H,I).

Some biogeographical studies have postulated that the coastal basins of the state of Maranhão represent a complex zone of endemism for freshwater fish [78,79]. Abreu et al. [79] concluded that the basins of the Parnaíba, Periá, Preguiças, Munim, Itapecuru, and Mearim rivers may represent an ecoregion distinct from that of the Turiaçu basin and the neighboring rivers, such as the Maracaçumé and Gurupi, and those of the western coastal zone. This conclusion is supported by the results of the present study. In this context, it is possible that further research focused on specific morphological traits will provide more conclusive support for the molecular entities identified as distinct taxonomic units.

The second case described here is the MOTU formed by *Pimelodus* sp. from the Munim basin in the state of Maranhão and *P. maculatus* from the basin of the São Francisco River, with a maximum intra-MOTU distance of 0.93% and minimum inter-MOTU divergence of 1.14%, from the *P. maculatus* MOTU of the basins of the Paraíba do Sul River, and the upper and lower Paraná (Figure 1, Table 1). The presence of the spots on the flanks of the *Pimelodus* sp. specimens (Figure 3G) collected from the Munim basin appears to confirm its morphological similarities with *Pimelodus maculatus* LaCepède, 1803.

These findings indicate a single taxonomic unit, which would amplify the known occurrence of *P. maculatus* to the basin of the Munim River. A number of points support this hypothesis, including the reduced intraspecific genetic divergence observed here, and the occurrence of *P. maculatus* reported from the Parnaíba basin [80,81,82]. Given the species composition, the dynamics of the local ecosystems, and their environmental conditions, the Parnaíba and Munim basins, together with the small coastal basins further east, should be considered to be a single ecoregion [83,84,85].

Only two congeners, *P. blochii* and *P. ornatus*, were recorded previously in the Munim basin [86,87,88,89,90,91], and *P. maculatus* was not registered in any of these studies. However, a greater sampling effort and a more detailed study would be necessary to better understand this scenario. Some cases of species being introduced into the hydrographic basins of Maranhão have been reported [24,89,91]. In the present study, four specimens were collected from the Preto River, in the municipality of São Benedito do Rio Preto, in the state of Maranhão, which corresponds to one of the localities sampled by Vieira et al. [91], in the most comprehensive survey ever conducted of the basin of the Munim River.

One MOTU also linked the nominal *P. albofasciatus* from the Tocantins River with *P. blochii* from different Amazonian basins (Figure 1). These two taxa share a number of morphological traits that may provoke errors of identification. *Pimelodus albofasciatus* was described by Mees [92] based on specimens from Suriname, who nevertheless considered this species to be similar to *P. blochii* and comparable with the Eigenmann [77] variety *B*. These species are distinguished based on differences in their morphology and ecology, and their apparent geographical isolation. Morphologically, *P. albofasciatus* has larger eyes than *P. blochii* (0.7–1.0 in bony interorbital versus 1.0–1.7) and shorter dorsal spine (shorter or equal in size to the length of the head versus equal or longer). Ecologically, while *P. albofasciatus* is found in minor rivers and lakes, *P. blochii* is found more frequently in the lower stretches and estuaries of major rivers [92].

Based on the results of the present study, it is possible to infer that specimens of at least two distinct taxa were collected from the Tocantins River, being identified here as *P. blochii* and *P.* cf. *albofasciatus*, based on minor morphological differences and the molecular delimitation (Figure 5, Supplementary Table S3). Even so, previous studies [6,12,93,94] have found that only six *Pimelodus* species—*P. halisodous*, *P. joannis*, *P. quadratus*, *P. stewarti*, *P. luciae*, and *P. speciosus—*are exclusive to the Tocantins River. Three other species are non-exclusive, including *P. tetramerus* (Tocantins and Tapajós rivers), and *P. blochii* and *P. ornatus*, which are amply distributed in the Amazon, Orinoco, upper Corantijn, and Sipaliwini basins [92,95].

In the present study, a genetic divergence threshold of 1.48% was adopted as the cutoff point for the delimitation of species, a lower criterion than that used typically for the molecular identification of fish species, i.e., 2% [29,33]. However, the threshold used here provides a starting point for the discrimination of *Pimelodus* taxa based on their genetic distances. It is important to note here that the use of any specific threshold of molecular divergence to delimit species should be considered cautiously and take the other data available on the study group into account, including its evolutionary history, ecology, and morphological and behavioral data [31,33].

## 5. Conclusions

The results of the present study highlight emphatically the need for a comprehensive taxonomic review of the genus *Pimelodus*. The lineages identified from the Parnaíba ecoregions, Amazonas Estuary and Coastal Drainages, *sensu* Abell et al. [85], may represent a much greater diversity of *Pimelodus* species than recognized currently, although a more detailed and extensive investigation will be needed to provide a more definitive classification. It would also be potentially useful to apply a population genetics approach to assess the genetic diversity within the study populations. The DNA barcode analysis conducted in the present study was an important step in the delimitation of the species of this fish group, and indicated a new occurrence for the basin of the Munim River, in the Brazilian state of Maranhão. In particular, the findings of the present study indicate the existence of a number of potentially new species, based on the results of the molecular methods of species delimitation, which may be fundamental to guide further taxonomic research that will help to better understand the diversity of the freshwater fish of the Neotropical region.

## Figures and Tables

**Table 1:** Genetic K2P (%) distances between the *Pimelodus* species recorded in the present study.

	Mean Intra-	Maximum Intra-	NN	Distance to NN
**Nominal Species**				
*P. albofasciatus*	0.00	0.00	*P. blochii*	0.00
*P. albicans*	0.00	0.00	*Pimelodus* sp.	2.84
*P. argenteus*	2.11	3.01	*P. blochii*	0.81
*P. blochii*	1.19	3.53	*P. maculatus/P. albofasciatus*	0.00
*P. crypticus*	0.07	0.22	*P. albicans*	6.19
*P. fur*	0.00	0.00	*P. albofasciatus*	3.51
*P. grosskopfii*	0.00	0.00	*P. crypticus*	6.95
*P. maculatus*	2.17	7.15	*P. blochii/Pimelodus* sp.	0.00
*P. ornatus*	2.89	6.17	*Pimelodus* sp.	13.1
*P. pictus*	2.47	4.97	*P. pohli*	9.66
*P. pohli*	0.49	0.98	*P. albofasciatus*	2.83
*Pimelodus* sp.	0.28	0.46	*P. maculatus*	0.00
*P. yuma*	0.00	0.00	*P. maculatus/P. blochii*	4.48
**Consensus MOTUs**				
*P. albicans* (MOTU 1)	0.00	0.00	*Pimelodus* sp.*/P. maculatus* MOTU 20	2.84
*P. fur* (MOTU 2)	0.00	0.00	*P. blochii/P.* cf. *albofasciatus* MOTU 09	3.51
*P. grosskopfii* (MOTU 3)	0.00	0.00	*P. crypticus* MOTU 05	6.95
*P. yuma* (MOTU 4)	0.00	0.00	*P. blochii/P.* cf. *maculatus* MOTU 19	4.48
*P. crypticus* (MOTU 5)	0.07	0.22	*P. albicans* MOTU 01	6.19
*P. pohli* (MOTU 6)	0.49	0.98	*P. blochii/P.* cf. *albofasciatus* MOTU 09	2.83
*P. ornatus* (MOTU 7)	0.07	0.15	*P. ornatus* MOTU 08	2.91
*P. ornatus* (MOTU 8)	-	-	*P. ornatus* MOTU 07	2.91
*P. blochii/P.* cf. *albofasciatus* (MOTU 9)	0.07	0.33	*P. blochii/P.* cf. *maculatus* MOTU 19	1.18
*P. maculatus* (MOTU 10)	0.20	0.98	*Pimelodus* sp.*/P. maculatus* MOTU 20	1.14
*P.* cf. *argenteus* (MOTU 11)	-	-	*P. blochii* MOTU 24	0.81
*P. pictus* (MOTU 12)	0.30	0.45	*P. pictus* MOTU 13	4.47
*P. pictus* (MOTU 13)	-	-	*P. pictus* MOTU 12	4.47
*P. blochii* (MOTU 14)	0.10	0.31	*P. blochii* MOTU 18	0.78
*P. ornatus* (MOTU 15)	0.02	0.15	*P. ornatus* MOTU 22	0.79
*P. ornatus* (MOTU 16)	0.09	0.31	*P. ornatus* MOTU 17	2.83
*P. ornatus* (MOTU 17)	0.32	0.65	*P. ornatus* MOTU 15	1.48
*P. blochii* (MOTU 18)	0.05	0.31	*P. blochii* MOTU 14	0.78
*P. blochii/P.* cf. *maculatus* (MOTU 19)	0.10	0.49	*P. blochii/P.* cf. *albofasciatus* MOTU 09	1.18
*Pimelodus* sp.*/P. maculatus* (MOTU 20)	0.27	0.93	*P. maculatus* MOTU 10	1.14
*P.* cf. *argenteus* (MOTU 21)	0.49	0.49	*P. blochii/P.* cf. *albofasciatus* MOTU 09	1.65
*P. ornatus* (MOTU 22)	-	-	*P. ornatus* MOTU 15	0.79
*P. blochii* (MOTU 23)	0.42	0.95	*P. blochii* MOTU 24	0.78
*P. blochii* (MOTU 24)	0.00	0.00	*P. blochii* MOTU 23	0.78

Mean and maximum intragroup distances, Nearest Neighbors (NN), and the minimum distance to the NN for the nominal species and the consensus MOTUs.

## Data Availability

The sequences included in the present study are available from GenBank (https://www.ncbi.nlm.nih.gov/genbank/, accessed on 16 January 2024). The voucher specimens used for the taxonomic diagnoses are deposited in the Fish Zoological Collection of the National Amazonian Research Institute (INPA) in Manaus, and the collections of the Museum of Zoology of the University of São Paulo (MZUSP), in São Paulo, and the Museum of Zoology of Londrina State University (MZUEL), in Londrina. The remaining specimens are held in the Molecular Biology Laboratory (LABMOL) of the GENBIMOL complex of the Caxias campus of Maranhão State University (UEMA). All the other data are contained in this manuscript.

## Supplementary Materials

The following supporting information can be downloaded at: https://www.mdpi.com/article/10.3390/biology13030162/s1, Table S1: The COI sequences generated in the present study; Table S2: The COI sequences obtained from GenBank; Table S3: Mean intra- and inter-MOTU genetic distances; Figure S1: Neighbour-Joining (NJ) Tree.
