# Supplementary material for: Molecular Evidence Reveals Taxonomic Uncertainties and Cryptic Diversity in the Neotropical Catfish of the Genus Pimelodus (Siluriformes: Pimelodidae)
Source: Biology (Basel). 2024 Mar 2;13(3):162. doi: 10.3390/biology13030162 (PMC10968110; doi:10.3390/biology13030162)
Supplement: Supplementary file 1 [file biology-13-00162-s001.zip › Table S1 COI sequences generated in this study.pdf]

Table S1. Collecting localities of the *Pimelodus* specimens obtained from the hydrographic basins of the Brazilian states of Maranhão, Piauí, and Tocantins.

Species (MOTU)	Code	River (Locality)	Basin	Museum voucher	GenBank
P. cf. *albofasciatus* (MOTU 9)	TO126	Tocantins (Imperatriz)	Tocantins-Araguaia	INPA-ICT 060050<br>Fig. 3 (H)	PP133038
P. cf. *albofasciatus* (MOTU 9)	TO127	Tocantins (Imperatriz)	Tocantins-Araguaia	INPA-ICT 060050	PP133039
P. blochii (MOTU 18)	ITA02	Itapecuru (Caxias)	Itapecuru	-	PP132870
P. blochii (MOTU 18)	ITA03	Itapecuru (Caxias)	Itapecuru	-	PP132871
P. blochii (MOTU 18)	ITA04	Itapecuru (Caxias)	Itapecuru	-	PP132872
P. blochii (MOTU 18)	ITA05	Itapecuru (Caxias)	Itapecuru	-	PP132873
P. blochii (MOTU 18)	ITA06	Itapecuru (Caxias)	Itapecuru	-	PP132874
P. blochii (MOTU 18)	ITA07	Itapecuru (Caxias)	Itapecuru	-	PP132875
P. blochii (MOTU 18)	ITA08	Itapecuru (Caxias)	Itapecuru	-	PP132876
P. blochii (MOTU 18)	ITA09	Itapecuru (Caxias)	Itapecuru	-	PP132877
P. blochii (MOTU 18)	ITA10	Itapecuru (Caxias)	Itapecuru	-	PP132878
P. blochii (MOTU 18)	ITA11	Itapecuru (Caxias)	Itapecuru	-	PP132879
P. blochii (MOTU 18)	ITA12	Itapecuru (Caxias)	Itapecuru	Fig. 2 (F)	PP132880
P. blochii (MOTU 18)	ITA13	Itapecuru (Caxias)	Itapecuru	-	PP132881
P. blochii (MOTU 18)	ITA14	Itapecuru (Caxias)	Itapecuru	-	PP132882
P. blochii (MOTU 18)	MDOU01	Itapecuru (Itapecuru Mirim)	Itapecuru	-	PP132883
P. blochii (MOTU 18)	MDOU02	Itapecuru (Itapecuru Mirim)	Itapecuru	-	PP132884
P. blochii (MOTU 18)	MDOU10	Itapecuru (Itapecuru Mirim)	Itapecuru	-	PP132885
P. blochii (MOTU 18)	MDOU16	Itapecuru (Itapecuru Mirim)	Itapecuru	-	PP132886
P. blochii (MOTU 18)	MDOU28	Itapecuru (Aldeias Altas)	Itapecuru	MZUSP 104570	PP132887
P. blochii (MOTU 18)	MDOU33	Itapecuru (Caxias)	Itapecuru	-	PP132888
P. blochii (MOTU 18)	MDOU35	Itapecuru (Caxias)	Itapecuru	-	PP132889
P. blochii (MOTU 18)	MEA43	Mearim (Pedreiras)	Mearim	-	PP132890
P. blochii (MOTU 18)	MEA44	Mearim (Pedreiras)	Mearim	-	PP132891
P. blochii (MOTU 18)	MEA45	Mearim (Pedreiras)	Mearim	-	PP132892
P. blochii (MOTU 18)	MEA46	Mearim (Pedreiras)	Mearim	-	PP132893

P. blochii (MOTU 18)	MEA47	Mearim (Pedreiras)	Mearim	-	PP132894
P. blochii (MOTU 18)	MEA49	Mearim (Pedreiras)	Mearim	-	PP132895
P. blochii (MOTU 18)	MEA459	Mearim (Barra do Corda)	Mearim	-	PP132896
P. blochii (MOTU 18)	MEA460	Mearim (Barra do Corda)	Mearim	MZUEL 15371	PP132897
P. blochii (MOTU 18)	MEA729	Mearim (Vitória do Mearim)	Mearim	-	PP132898
P. blochii (MOTU 18)	MEA730	Mearim (Vitória do Mearim)	Mearim	-	PP132899
P. blochii (MOTU 18)	MEA731	Mearim (Vitória do Mearim)	Mearim	Fig. 2 (A)	PP132900
P. blochii (MOTU 18)	MEA732	Mearim (Vitória do Mearim)	Mearim	MZUEL 15372	PP132901
P. blochii (MOTU 18)	MEA735	Mearim (Vitória do Mearim)	Mearim	-	PP132902
P. blochii (MOTU 18)	MEA736	Mearim (Vitória do Mearim)	Mearim	-	PP132903
P. blochii (MOTU 18)	MDOU52	Mearim	Mearim	-	PP132904
P. blochii (MOTU 18)	MDOU54	Mearim	Mearim	-	PP132905
P. blochii (MOTU 18)	MDOU55	Mearim	Mearim	-	PP132906
P. blochii (MOTU 18)	COR112	Corda (Barra do Corda)	Mearim	-	PP132907
P. blochii (MOTU 18)	COR113	Corda (Barra do Corda)	Mearim	-	PP132908
P. blochii (MOTU 18)	COR115	Corda (Barra do Corda)	Mearim	-	PP132909
P. blochii (MOTU 18)	COR116	Corda (Barra do Corda)	Mearim	Fig. 2 (E)	PP132910
P. blochii (MOTU 18)	FLOR112	Flores (Joselândia)	Mearim	-	PP132911
P. blochii (MOTU 18)	FLOR116	Flores (Joselândia)	Mearim	-	PP132912
P. blochii (MOTU 18)	FLOR117	Flores (Joselândia)	Mearim	-	PP132913
P. blochii (MOTU 18)	FLOR118	Flores (Joselândia)	Mearim	Fig. 2 (D)	PP132914
P. blochii (MOTU 18)	FLOR124	Flores (Joselândia)	Mearim	-	PP132915
P. blochii (MOTU 18)	FLOR125	Flores (Joselândia)	Mearim	-	PP132916
P. blochii (MOTU 18)	FLOR128	Flores (Joselândia)	Mearim	-	PP132917
P. blochii (MOTU 18)	FLOR130	Flores (Joselândia)	Mearim	-	PP132918
P. blochii (MOTU 18)	FLOR131	Flores (Joselândia)	Mearim	-	PP132919
P. blochii (MOTU 18)	FLOR132	Flores (Joselândia)	Mearim	-	PP132920
P. blochii (MOTU 18)	FLOR134	Flores (Joselândia)	Mearim	-	PP132921

P. blochii (MOTU 18)	GRA64	Grajaú (Grajaú)	Mearim	-	PP132922
P. blochii (MOTU 18)	GRA65	Grajaú (Grajaú)	Mearim	-	PP132923
P. blochii (MOTU 18)	GRA66	Grajaú (Grajaú)	Mearim	-	PP132924
P. blochii (MOTU 18)	GRA67	Grajaú (Grajaú)	Mearim	Fig. 2 (C)	PP132925
P. blochii (MOTU 18)	GRA68	Grajaú (Grajaú)	Mearim	-	PP132926
P. blochii (MOTU 18)	GRA69	Grajaú (Grajaú)	Mearim	-	PP132927
P. blochii (MOTU 18)	GRA70	Grajaú (Grajaú)	Mearim	-	PP132928
P. blochii (MOTU 18)	GRA71	Grajaú (Grajaú)	Mearim	-	PP132929
P. blochii (MOTU 18)	GRA72	Grajaú (Grajaú)	Mearim	-	PP132930
P. blochii (MOTU 18)	GRA73	Grajaú (Grajaú)	Mearim	-	PP132931
P. blochii (MOTU 18)	GRA74	Grajaú (Grajaú)	Mearim	-	PP132932
P. blochii (MOTU 18)	GRA75	Grajaú (Grajaú)	Mearim	-	PP132933
P. blochii (MOTU 18)	GRA76	Grajaú (Grajaú)	Mearim	-	PP132934
P. blochii (MOTU 18)	GRA79	Grajaú (Grajaú)	Mearim	-	PP132935
P. blochii (MOTU 18)	MDOU68	Pindaré	Mearim	-	PP132936
P. blochii (MOTU 18)	MDOU69	Pindaré	Mearim	-	PP132937
P. blochii (MOTU 18)	MDOU70	Pindaré	Mearim	-	PP132938
P. blochii (MOTU 18)	MDOU71	Pindaré	Mearim	-	PP132939
P. blochii (MOTU 18)	MDOU73	Pindaré	Mearim	-	PP132940
P. blochii (MOTU 18)	MDOU74	Pindaré	Mearim	-	PP132941
P. blochii (MOTU 18)	MDOU75	Pindaré	Mearim	-	PP132942
P. blochii (MOTU 18)	MDOU77	Pindaré	Mearim	-	PP132943
P. blochii (MOTU 18)	MDOU78	Pindaré	Mearim	-	PP132944
P. blochii (MOTU 18)	PIN116	Pindaré (Pindaré Mirim)	Mearim	-	PP132945
P. blochii (MOTU 18)	PIN117	Pindaré (Pindaré Mirim)	Mearim	-	PP132946
P. blochii (MOTU 18)	PIN247	Pindaré (Pindaré Mirim)	Mearim	Fig. 2 (B)	PP132947
P. blochii (MOTU 18)	PIN248	Pindaré (Pindaré Mirim)	Mearim	MZUEL17394	PP132948
P. blochii (MOTU 18)	PIN250	Pindaré (Pindaré Mirim)	Mearim	-	PP132949

P. blochii (MOTU 18)	PIN251	Pindaré (Pindaré Mirim)	Mearim	-	PP132950
P. blochii (MOTU 18)	PIN252	Pindaré (Pindaré Mirim)	Mearim	-	PP132951
P. blochii (MOTU 14)	PARN47	Parnaíba (Coelho Neto)	Parnaíba	-	PP132952
P. blochii (MOTU 14)	PARN48	Parnaíba (Coelho Neto)	Parnaíba	-	PP132953
P. blochii (MOTU 14)	PARN49	Parnaíba (Coelho Neto)	Parnaíba	-	PP132954
P. blochii (MOTU 14)	PARN51	Parnaíba (Coelho Neto)	Parnaíba	-	PP132955
P. blochii (MOTU 14)	PARN52	Parnaíba (Coelho Neto)	Parnaíba	-	PP132956
P. blochii (MOTU 14)	PARN53	Parnaíba (Coelho Neto)	Parnaíba	-	PP132957
P. blochii (MOTU 14)	PARN54	Parnaíba (Coelho Neto)	Parnaíba	-	PP132958
P. blochii (MOTU 14)	PARN56	Parnaíba (Coelho Neto)	Parnaíba	Fig. 2 (G)	PP132959
P. blochii (MOTU 14)	PARN57	Parnaíba (Coelho Neto)	Parnaíba	-	PP132960
P. blochii (MOTU 14)	PARN58	Parnaíba (Coelho Neto)	Parnaíba	-	PP132961
P. blochii (MOTU 14)	PARN60	Parnaíba (Coelho Neto)	Parnaíba	-	PP132962
P. blochii (MOTU 14)	PARN61	Parnaíba (Coelho Neto)	Parnaíba	-	PP132963
P. blochii (MOTU 14)	PARN62	Parnaíba (Coelho Neto)	Parnaíba	-	PP132964
P. blochii (MOTU 14)	PARN63	Parnaíba (Coelho Neto)	Parnaíba	-	PP132965
P. blochii (MOTU 14)	PARN64	Parnaíba (Coelho Neto)	Parnaíba	-	PP132966
P. blochii (MOTU 19)	TUR99	Turiaçu (Santa Helena)	Turiaçu	-	PP132967
P. blochii (MOTU 19)	TUR100	Turiaçu (Santa Helena)	Turiaçu	-	PP132968
P. blochii (MOTU 19)	TUR101	Turiaçu (Santa Helena)	Turiaçu	Fig. 2 (H)	PP132969
P. blochii (MOTU 19)	TUR102	Turiaçu (Santa Helena)	Turiaçu	MZUEL17437	PP132970
P. blochii (MOTU 19)	TUR103	Turiaçu (Santa Helena)	Turiaçu	-	PP132971
P. blochii (MOTU 19)	TUR343	Turiaçu (Santa Helena)	Turiaçu	-	PP132972
P. blochii (MOTU 19)	TUR347	Turiaçu (Santa Helena)	Turiaçu	-	PP132973
P. blochii (MOTU 23)	TUR114	Turiaçu (Santa Helena)	Turiaçu	MZUEL17437	PP132974
P. blochii (MOTU 23)	TUR175	Turiaçu (Santa Helena)	Turiaçu	Fig. 2 (I)	PP132975
P. blochii (MOTU 23)	TUR177	Turiaçu (Santa Helena)	Turiaçu	-	PP132976
P. blochii (MOTU 23)	TUR178	Turiaçu (Santa Helena)	Turiaçu	-	PP132977

P. blochii (MOTU 23)	TUR179	Turiaçu (Santa Helena)	Turiaçu	-	PP132978
P. blochii (MOTU 23)	TUR345	Turiaçu (Santa Helena)	Turiaçu	-	PP132979
P. blochii (MOTU 23)	TUR346	Turiaçu (Santa Helena)	Turiaçu	-	PP132980
P. blochii (MOTU 24)	TO268/278	Tocantins (Babaçulândia)	Tocantins-Araguaia	INPA-ICT059981	PP132981
P. blochii (MOTU 24)	TO296/298	Tocantins (Babaçulândia)	Tocantins-Araguaia	INPA-ICT059981	PP132982
P. blochii (MOTU 24)	TO297	Tocantins (Babaçulândia)	Tocantins-Araguaia	INPA-ICT059981	PP132983
P. blochii (MOTU 24)	TO299	Tocantins (Babaçulândia)	Tocantins-Araguaia	INPA-ICT059981 Fig. 2 (J)	PP132984
P. blochii (MOTU 24)	TO623	Tocantins (Babaçulândia)	Tocantins-Araguaia	INPA-ICT060010	PP132985
P. blochii (MOTU 24)	TO624	Tocantins (Babaçulândia)	Tocantins-Araguaia	INPA-ICT060010	PP132986
P. blochii (MOTU 24)	TO625	Tocantins (Babaçulândia)	Tocantins-Araguaia	INPA-ICT060010	PP132987
P. blochii (MOTU 24)	TO626	Tocantins (Babaçulândia)	Tocantins-Araguaia	INPA-ICT060010	PP132988
P. ornatus (MOTU 7)	ITA17	Itapecuru (Caxias)	Itapecuru	-	PP132989
P. ornatus (MOTU 7)	ITA18	Itapecuru (Caxias)	Itapecuru	-	PP132990
P. ornatus (MOTU 7)	ITA19	Itapecuru (Caxias)	Itapecuru	Fig. 3 (A)	PP132991
P. ornatus (MOTU 7)	ITA20	Itapecuru (Caxias)	Itapecuru	-	PP132992
P. ornatus (MOTU 7)	ITA21	Itapecuru (Caxias)	Itapecuru	-	PP132993
P. ornatus (MOTU 7)	ITA22	Itapecuru (Caxias)	Itapecuru	-	PP132994
P. ornatus (MOTU 7)	MSAC01	Itapecuru (Itapecuru Mirim)	Itapecuru	-	PP132995
P. ornatus (MOTU 7)	MSAC04	Itapecuru (Itapecuru Mirim)	Itapecuru	-	PP132996
P. ornatus (MOTU 7)	MSAC06	Itapecuru (Itapecuru Mirim)	Itapecuru	-	PP132997
P. ornatus (MOTU 7)	MSAC07	Itapecuru (Caxias)	Itapecuru	MZUSP 104572	PP132998
P. ornatus (MOTU 7)	MSAC11	Itapecuru (Aldeias Altas)	Itapecuru	MZUSP104565	PP132999
P. ornatus (MOTU 7)	MSAC12	Itapecuru (Colinas)	Itapecuru	-	PP133000
P. ornatus (MOTU 7)	MSAC13	Itapecuru (Colinas)	Itapecuru	-	PP133001
P. ornatus (MOTU 7)	MSAC14	Itapecuru (Colinas)	Itapecuru	-	PP133002
P. ornatus (MOTU 7)	GRA80	Grajaú (Grajaú)	Mearim	Fig. 3 (D)	PP133003
P. ornatus (MOTU 7)	MEA513	Mearim (Barra do Corda)	Mearim	-	PP133004

P. ornatus (MOTU 7)	MEA514	Mearim (Barra do Corda)	Mearim	-	PP133005
P. ornatus (MOTU 7)	MEA1044	Mearim (Pedreiras)	Mearim	-	PP133006
P. ornatus (MOTU 7)	MEA1045	Mearim (Pedreiras)	Mearim	Fig. 3 (B)	PP133007
P. ornatus (MOTU 7)	MSAC15	Pindaré	Mearim	-	PP133008
P. ornatus (MOTU 7)	MSAC17	Pindaré	Mearim	-	PP133009
P. ornatus (MOTU 7)	MSAC18	Pindaré	Mearim	-	PP133010
P. ornatus (MOTU 7)	MSAC20	Pindaré	Mearim	-	PP133011
P. ornatus (MOTU 7)	MSAC22	Pindaré	Mearim	-	PP133012
P. ornatus (MOTU 7)	MSAC23	Pindaré	Mearim	-	PP133013
P. ornatus (MOTU 7)	MSAC24	Pindaré	Mearim	-	PP133014
P. ornatus (MOTU 7)	MSAC25	Pindaré	Mearim	-	PP133015
P. ornatus (MOTU 7)	MSAC26	Pindaré	Mearim	-	PP133016
P. ornatus (MOTU 7)	MSAC28	Pindaré	Mearim	-	PP133017
P. ornatus (MOTU 7)	MSAC29	Pindaré	Mearim	-	PP133018
P. ornatus (MOTU 7)	MSAC30	Pindaré	Mearim	-	PP133019
P. ornatus (MOTU 7)	MSAC31	Pindaré	Mearim	-	PP133020
P. ornatus (MOTU 7)	MSAC33	Pindaré	Mearim	-	PP133021
P. ornatus (MOTU 7)	PIN91	Pindaré (Pindaré Mirim)	Mearim	-	PP133022
P. ornatus (MOTU 7)	PIN92	Pindaré (Pindaré Mirim)	Mearim	MZUEL 17395<br>Fig. 3 (C)	PP133023
P. ornatus (MOTU 7)	PIN93	Pindaré (Pindaré Mirim)	Mearim	-	PP133024
P. ornatus (MOTU 15)	TUR96	Turiaçu (Santa Helena)	Turiaçu	-	PP133025
P. ornatus (MOTU 15)	TUR97	Turiaçu (Santa Helena)	Turiaçu	MZUEL 17439	PP133026
P. ornatus (MOTU 15)	TUR98	Turiaçu (Santa Helena)	Turiaçu	-	PP133027
P. ornatus (MOTU 15)	TUR180	Turiaçu (Santa Helena)	Turiaçu	Fig. 3 (F)	PP133028
P. ornatus (MOTU 15)	TUR181	Turiaçu (Santa Helena)	Turiaçu	-	PP133029
P. ornatus (MOTU 15)	TUR182	Turiaçu (Santa Helena)	Turiaçu	-	PP133030
P. ornatus (MOTU 15)	TUR183	Turiaçu (Santa Helena)	Turiaçu	-	PP133031

P. ornatus (MOTU 15)	TUR184	Turiaçu (Santa Helena)	Turiaçu	-	PP133032
P. ornatus (MOTU 15)	TUR185	Turiaçu (Santa Helena)	Turiaçu	-	PP133033
P. ornatus (MOTU 15)	TUR348	Turiaçu (Santa Helena)	Turiaçu	-	PP133034
P. ornatus (MOTU 15)	TUR349	Turiaçu (Santa Helena)	Turiaçu	-	PP133035
P. ornatus (MOTU 15)	TUR350	Turiaçu (Santa Helena)	Turiaçu	-	PP133036
P. ornatus (MOTU 8)	PARN80	Paranaíba (Coelho Neto)	Parnaíba	Fig. 3 (E)	PP133037
Pimelodus sp. (MOTU 20)	MUN67	Preto (São Benedito do rio Preto)	Munim	-	PP133040
Pimelodus sp. (MOTU 20)	MUN68	Preto (São Benedito do rio Preto)	Munim	-	PP133041
Pimelodus sp. (MOTU 20)	MUN69	Preto (São Benedito do rio Preto)	Munim	Fig. 3 (G)	PP133042
Pimelodus sp. (MOTU 20)	MUN70	Preto (São Benedito do rio Preto)	Munim	-	PP133043

Code = field number
