# Supplementary material for: Molecular Evidence Reveals Taxonomic Uncertainties and Cryptic Diversity in the Neotropical Catfish of the Genus Pimelodus (Siluriformes: Pimelodidae)
Source: Biology (Basel). 2024 Mar 2;13(3):162. doi: 10.3390/biology13030162 (PMC10968110; doi:10.3390/biology13030162)
Supplement: Supplementary file 1 [file biology-13-00162-s001.zip › Table S2 COI sequences obtained from Genbank.pdf]

Table S2. Details and GenBank accession numbers of the samples of the *Pimelodus* specimens included in the analyses presented here.

Species (MOTU)	River (Locality)	Basin	GenBank	Author
P. albicans (MOTU 1)	Baixo Paraná (Rosario)	Paraná	KU288929	Diaz et al. (2016)
P. albicans (MOTU 1)	Planície do Pampa	La Prata	JX111817	Rosso et al. (2012)
P. cf. *argenteus* (MOTU 21)	Paraguai	Paraguai	KP294270	Lima et al. (2016)
P. cf. *argenteus* (MOTU 21)	Paraguai	Paraguai	KP294271	Lima et al. (2016)
P. cf. *argenteus* (MOTU 11)	Paraguai	Paraguai	KP294272	Lima et al. (2016)
P. blochii (MOTU 9)	Vaupés	Amazonas	OK206796	Martinez et al. (2022)
P. blochii (MOTU 9)	Vaupés	Amazonas	OK206797	Martinez et al. (2022)
P. blochii (MOTU 9)	Vaupés	Amazonas	OK206798	Martinez et al. (2022)
P. blochii (MOTU 9)	Napo	Amazonas	KT952419	Garcia-Davila et al. (2015)
P. blochii (MOTU 9)	Negro	Amazonas	FJ978039	Ardura et al. (2010)
P. crypticus (MOTU 5)	Alto Cauca	Magdalena	OK206805	Martinez et al. (2022)
P. crypticus (MOTU 5)	Alto Cauca	Magdalena	OK206806	Martinez et al. (2022)
P. crypticus (MOTU 5)	Alto Cauca	Magdalena	OK206807	Martinez et al. (2022)
P. crypticus (MOTU 5)	Alto Cauca	Magdalena	OK206808	Martinez et al. (2022)
P. crypticus (MOTU 5)	Alto Cauca	Magdalena	OK206809	Martinez et al. (2022)
P. crypticus (MOTU 5)	Alto Cauca	Magdalena	OK206810	Martinez et al. (2022)
P. fur (MOTU 2)	São Francisco	São Francisco	HM404958	Carvalho et al. (2011)
P. fur (MOTU 2)	São Francisco	São Francisco	HM405192	Carvalho et al. (2011)
P. fur (MOTU 2)	São Francisco	São Francisco	HM405197	Carvalho et al. (2011)
P. grosskopfii (MOTU 3)	Cauca	Magdalena	OK206726	Martinez et al. (2022)
P. grosskopfii (MOTU 3)	Cauca	Magdalena	OK206727	Martinez et al. (2022)
P. grosskopfii (MOTU 3)	Cauca	Magdalena	OK206728	Martinez et al. (2022)
P. grosskopfii (MOTU 3)	Cauca	Magdalena	OK206729	Martinez et al. (2022)
P. grosskopfii (MOTU 3)	Cauca	Magdalena	OK206730	Martinez et al. (2022)
P. maculatus (MOTU 10)	Cinzas	Alto Paraná	KM897670	Frantine-Silva et al. (2015)
P. maculatus (MOTU 10)	Cinzas	Alto Paraná	KM897662	Frantine-Silva et al. (2015)

P. maculatus (MOTU 10)	Cinzas	Alto Paraná	KM897661	Frantine-Silva et al. (2015)
P. maculatus (MOTU 10)	Paranapanema (Canoas I)	Alto Paraná	KM897672	Frantine-Silva et al. (2015)
P. maculatus (MOTU 10)	Paranapanema (Canoas I)	Alto Paraná	KM897666	Frantine-Silva et al. (2015)
P. maculatus (MOTU 10)	Paranapanema (Canoas I)	Alto Paraná	KM897660	Frantine-Silva et al. (2015)
P. maculatus (MOTU 10)	Paranapanema (Canoas I)	Alto Paraná	KM897658	Frantine-Silva et al. (2015)
P. maculatus (MOTU 10)	Paranapanema (Canoas II)	Alto Paraná	KM897667	Frantine-Silva et al. (2015)
P. maculatus (MOTU 10)	Paranapanema (Canoas II)	Alto Paraná	KM897663	Frantine-Silva et al. (2015)
P. maculatus (MOTU 10)	Paranapanema (Canoas II)	Alto Paraná	KM897657	Frantine-Silva et al. (2015)
P. maculatus (MOTU 10)	Paranapanema (Canoas II)	Alto Paraná	KM897656	Frantine-Silva et al. (2015)
P. maculatus (MOTU 10)	Paranapanema, (Canoas I)	Alto Paraná	KM897654	Frantine-Silva et al. (2015)
P. maculatus (MOTU 10)	Paraná	Ato Paraná	MF595253	Calegari et al. (2019)
P. maculatus (MOTU 10)	Paraná	Ato Paraná	GU701570	Pereira et al. (2013)
P. maculatus (MOTU 10)	Paraná	Ato Paraná	GU701571	Pereira et al. (2013)
P. maculatus (MOTU 10)	Paraná	Ato Paraná	JN989127	Pereira et al. (2013)
P. maculatus (MOTU 10)	Paraná (Rosario)	Baixo Paraná	KU288802	Diaz et al. (2016)
P. maculatus (MOTU 10)	Paraíba do Sul	Paraíba do Sul	GU702297	Pereira et al. (2011)
P. maculatus (MOTU 10)	Paraíba do Sul	Paraíba do Sul	GU702298	Pereira et al. (2011)
P. maculatus (MOTU 10)	Paraíba do Sul	Paraíba do Sul	GU702299	Pereira et al. (2011)
P. maculatus (MOTU 10)	Paraíba do Sul	Paraíba do Sul	GU702300	Pereira et al. (2011)
P. maculatus (MOTU 10)	Paraíba do Sul	Paraíba do Sul	GU702301	Pereira et al. (2011)
P. maculatus (MOTU 20)	São Francisco	São Francisco	HM405191	Carvalho et al. (2011)
P. maculatus (MOTU 20)	São Francisco	São Francisco	HM405190	Carvalho et al. (2011)
P. maculatus (MOTU 20)	São Francisco	São Francisco	HM405189	Carvalho et al. (2011)
P. maculatus (MOTU 20)	São Francisco	São Francisco	HM405034	Carvalho et al. (2011)
P. maculatus (MOTU 20)	São Francisco	São Francisco	HM405034	Carvalho et al. (2011)
P. maculatus (MOTU 20)	São Francisco	São Francisco	NC_032695	Resende et al. (2016)
P. maculatus (MOTU 20)	São Francisco	São Francisco	KX371345	Resende et al. (2016)
P. cf. *maculatus* (MOTU 19)	Paraguai	Paraguai	KP294274	Lima et al. (2016)
P. cf. *maculatus* (MOTU 19)	Paraguai	Paraguai	KP294275	Lima et al. (2016)
P. cf. *maculatus* (MOTU 19)	Paraguai	Paraguai	KP294276	Lima et al. (2016)

P. cf. *maculatus* (MOTU 19)	Paraguai	Paraguai	KP294277	Lima et al. (2016)
P. cf. *maculatus* (MOTU 19)	Paraguai	Paraguai	KP294278	Lima et al. (2016)
P. ornatus (MOTU 16)	Maroni	Maroni	MZ052050	Papa et al. (2021)
P. ornatus (MOTU 16)	Maroni	Maroni	MZ052031	Papa et al. (2021)
P. ornatus (MOTU 16)	Maroni	Maroni	MZ051469	Papa et al. (2021)
P. ornatus (MOTU 16)	Maroni	Maroni	MZ051391	Papa et al. (2021)
P. ornatus (MOTU 16)	Maroni	Maroni	MZ051341	Papa et al. (2021)
P. ornatus (MOTU 16)	Maroni	Maroni	MZ050914	Papa et al. (2021)
P. ornatus (MOTU 16)	Maroni	Maroni	MZ050900	Papa et al. (2021)
P. ornatus (MOTU 17)	Paraguai	Paraguai	KP294265	Lima et al. (2016)
P. ornatus (MOTU 17)	Paraguai	Paraguai	KP294266	Lima et al. (2016)
P. ornatus (MOTU 17)	Paraguai	Paraguai	KP294267	Lima et al. (2016)
P. ornatus (MOTU 17)	Paraguai	Paraguai	KP294268	Lima et al. (2016)
P. ornatus (MOTU 17)	Paraguai	Paraguai	KP294269	Lima et al. (2016)
P. ornatus (MOTU 22)	Marañon	Amazonas	KT952422	Garcia-Davila et al. (2015)
P. pictus (MOTU 12)	Orinoco	Orinoco	OK206793	Martinez et al. (2022)
P. pictus (MOTU 12)	Orinoco	Orinoco	OK206794	Martinez et al. (2022)
P. pictus (MOTU 12)	Orinoco	Orinoco	OK206795	Martinez et al. (2022)
P. pictus (MOTU 13)	Amazonas	Amazonas	KT952417	Garcia-Davila et al. (2015)
P. pohli MOTU 6)	São Francisco	São Francisco	HM405196	Carvalho et al. (2011)
P. pohli MOTU 6)	São Francisco	São Francisco	HM405195	Carvalho et al. (2011)
P. pohli MOTU 6)	São Francisco	São Francisco	HM405194	Carvalho et al. (2011)
P. pohli MOTU 6)	São Francisco	São Francisco	HM405193	Carvalho et al. (2011)
P. yuma (MOTU 4)	Cauca	Magdalena	OK206775	Martinez et al., 2022
P. yuma (MOTU 4)	Cauca	Magdalena	OK206774	Martinez et al. (2022)
P. yuma (MOTU 4)	Cauca	Magdalena	OK206772	Martinez et al. (2022)
P. yuma (MOTU 4)	Cauca	Magdalena	OK206771	Martinez et al. (2022)

Referências

Ardura, A, Linde AR, Moreira JC e Garcia-Vazquez E DNA barcoding for conservation and management of Amazonian commercial fish. *Biological Conservation* **2010**, 143, 1438-1443, https://doi.org/10.1016/j.biocon.2010.03.019.

Calegari, B.B.; Vari, R.P.; Reis, R.E. Phylogenetic systematics of the driftwood catfishes (Siluriformes: Auchenipteridae): a combined morphological and molecular analysis. *Zool J Linn Soc* **2019**, 187, 661-773, https://doi.org/10.1093/zoolinnean/zlz036.

Carvalho, D.C.; Oliveira, D.A.A.; Pompeu, O.S.; Leal, C.G.; Oliveira, C.; Hanner, R. Deep barcode divergence in Brazilian freshwater fishes: the case of the São Francisco River basin. Mitochondrial DNA 2011, 22, 80–86, doi: 10.3109/19401736.2011.588214.

Díaz, J.; Villanova, G.V.; Brancolini, F.; Del Pazo, F.; Posner, V.M.; Grimberg, A.; Arranz, S.E. First DNA barcode reference library for the identification of South American freshwater fish from the lower Paraná river. PLoS One **2016**, 11, e0157419, https://doi.org/10.1371/journal.pone.0157419.

Frantine-Silva W, Sofia SH, Orsi ML e Almeida FS. DNA barcoding of freshwater ichthyoplankton in the Neotropics as a tool for ecological monitoring. Mol Ecol Resour 2015, 15, 1226-1237, https://doi.org/10.1111/1755-0998.12385.

García-Dávila, C.; Castro-Ruiz, D.; Renno, J.F.; Chota-Macuyama, W.; Carvajal-Vallejos, F.M.; Sanchez, H.; Ângulo, C.; Nolorbe, C.; Alvarado, J.; Estivals, G.; et al. Using barcoding of larvae for investigating the breeding seasons of Pimelodid catfishes from the Marañon, Napo and Ucayali rivers in the Peruvian Amazon. J Appl Ichthyol 2015, 31, 40-51, https://doi.org/10.1111/jai.12987.

Lima, T.P.C.; Egito, A.A.; Feijó, G.L.D.; Mauro R. A.; Ferraz, A.L.J. Molecular identification and phylogenetic analysis of Siluriformes from the Paraguay River basin, Brazil. Mitochondrial DNA Part A 2016, 28, 536-543, https://doi.org/10.3109/24701394.2016.1149825.

Martínez, J.G.; Rangel-Medrano, J.D.; Yepes-Acevedo, A.J.; Restrepo-Escobar, N.; Márquez, E.J. Species limits and introgression in *Pimelodus* from the Magdalena-Cauca River basin. Mol Phylogenet Evol 2022, 173, 107517, https://doi.org/10.1016/j.ympev.2022.107517.

Papa, Y.; Le Bail P.Y.; Covain, R. Genetic landscape clustering of a large DNA barcoding data set reveals shared patterns of genetic divergence among freshwater fishes of the Maroni Basin. Mol Ecol Resour, 2021, 21, 2109-2124, https://doi.org/10.1111/1755-0998.13402.

Pereira, L.H.; Maia, G.M.; Hanner, R.; Foresti, F.; Oliveira, C. DNA barcodes discriminate freshwater fishes from the Paraíba do Sul River Basin, São Paulo, Brazil. Mitochondrial DNA 2011, 22, 71-79, https://doi.org/10.3109/19401736.2010.532213.

Pereira, L.H.G.; Hanner, R.; Foresti, F.; Oliveira, C. Can DNA barcoding accurately discriminate megadiverse Neotropical freshwater fish fauna? BMC Genetics 2013, 14, 1471-2156, http://dx.doi.org/10.1186/1471-2156-14-20.

Resende, L.C.; Carmo, A.O.D.; Núñez-Rodriguez, D.; Pimentel, J.D.S.M.; Bedore, A.G.; Leal, H.G.; Kalapothakis, E. *Pimelodus maculatus* (Siluriformes, Pimelodidae): complete mtDNA sequence of an economically important fish from the São Francisco river basin. Mitochondrial DNA 2016, 1, 806-808, doi: 10.1080/23802359.2016.1219646.

Rosso, J.J.; Mabragaña, E.; Castro, G.; M.; Díaz de Astarloa, J.M. DNA barcoding Neotropical fishes: recent advances from the Pampa Plain, Argentina. Mol Ecol Resour 2012, 12, 999-1011, https://doi.org/10.1111/1755-0998.12010.
