# Supplementary material for: Molecular Evidence Reveals Taxonomic Uncertainties and Cryptic Diversity in the Neotropical Catfish of the Genus Pimelodus (Siluriformes: Pimelodidae)
Source: Biology (Basel). 2024 Mar 2;13(3):162. doi: 10.3390/biology13030162 (PMC10968110; doi:10.3390/biology13030162)
Supplement: Supplementary file 1 [file biology-13-00162-s001.zip › Table S3 Mean intra- and inter-MOTUS genetic distance.pdf]

Table S3. The mean genetic K2P distances (%) between the *Pimelodus* MOTUs. The mean intra-MOTU K2P (%) distances are shown in bold script in the diagonal.

	Species (MOTU)	1	2	3	4	5	6	7	8	9	10	11	12	13	14	15	16	17	18	19	20	21	22	23	24
1.	*P. ornatus* (MOTU 7)	**0.07**																							
2.	*P. ornatus* (MOTU 8)	03.15	**N/C**																						
3.	*P. ornatus* (MOTU 15)	05.55	05.13	**0.02**																					
4.	*P. ornatus* (MOTU 16)	04.28	03.87	04.05	**0.09**																				
5.	*P. ornatus* (MOTU 22)	06.07	05.64	00.81	04.56	**N/C**																			
6.	*P. ornatus* (MOTU 17)	05.01	04.19	01.70	03.04	02.19	**0.32**																		
7.	*P. blochii*/ *P.* cf. *albofasciatus* (MOTU 9)	17.56	16.36	17.51	17.86	17.75	17.75	**0.07**																	
8.	*P. blochii* (MOTU 18)	15.70	14.99	16.47	17.16	16.91	17.13	02.59	**0.05**																
9.	*P. blochii* (MOTU 14)	15.29	14.69	16.03	16.73	16.46	16.67	02.66	01.17	**0.10**															
10.	*P. blochii*/ *P.* cf. *maculatus* (MOTU 19)	16.04	14.90	16.77	16.96	17.20	16.77	01.69	02.49	02.70	**0.10**														
11.	*P. blochii* (MOTU 23)	16.14	15.01	16.30	16.28	16.73	16.73	02.36	02.90	02.52	02.88	**0.42**													
12.	*P. blochii* (MOTU 24)	16.28	15.12	16.63	16.67	17.05	16.35	01.84	02.66	02.26	02.14	01.15	**0.00**												
13.	*Pimelodus* sp./ *P. maculatus* (MOTU 20)	15.11	13.80	14.51	15.29	14.93	14.76	05.53	06.33	06.25	05.77	06.25	06.05	**0.27**											
14.	*P. maculatus* (MOTU 10)	16.26	14.67	15.52	16.15	15.94	15.59	06.45	07.28	07.21	06.69	06.82	06.94	01.49	**0.20**										
15.	*P.* cf. *argenteus* (MOTU 11)	16.51	15.76	16.42	16.84	16.84	16.03	01.74	02.67	02.26	02.16	01.64	00.99	06.01	06.91	**N/C**									
16.	*P.* cf. *argenteus* (MOTU 21)	16.42	15.88	17.43	17.41	17.85	17.03	01.94	03.27	03.55	01.96	03.76	02.94	06.58	07.52	02.93	**0.49**								
17.	*P. yuma* (MOTU 4)	15.55	15.55	16.75	16.75	16.75	16.69	05.70	05.96	06.63	04.69	05.39	05.47	05.96	06.42	05.22	05.74	**0.00**							
18.	*P. grosskopfii* (MOTU 3)	21.03	19.10	20.38	20.70	20.70	19.23	09.57	10.62	11.01	10.30	10.89	10.60	10.73	11.14	10.33	10.48	11.69	**0.00**						
19.	*P. crypticus* (MOTU 5)	20.08	19.76	20.08	20.40	20.40	18.93	08.08	09.58	09.78	09.06	09.37	09.09	09.63	10.65	8.82	09.64	10.15	07.16	**0.07**					
20.	*P. pictus* (MOTU 12)	19.20	17.33	17.84	17.39	18.15	17.78	11.74	13.98	14.09	12.22	12.42	12.53	12.59	13.00	12.25	13.12	12.41	15.66	13.55	**0.30**				
21.	*P. pictus* (MOTU 13)	17.04	15.26	15.28	16.29	15.05	15.70	11.45	11.23	11.33	10.33	10.27	11.08	10.64	10.85	10.83	11.95	11.41	15.14	14.80	04.64	**N/C**			
22.	*P. fur* (MOTU 2)	16.47	15.30	16.81	16.36	17.23	16.44	03.69	05.09	05.03	04.37	04.71	04.78	04.94	05.46	04.75	04.49	05.72	09.25	07.77	12.53	11.46	**0.00**		
23.	*P. pohli* (MOTU 6)	16.13	15.39	16.37	16.46	16.78	16.41	03.45	05.01	04.68	03.43	03.93	03.72	05.79	06.27	03.61	03.71	05.10	12.41	10.58	10.38	09.81	04.94	**0.49**	
24.	*P. albicans* (MOTU 1)	17.21	16.00	16.23	16.66	16.66	16.23	03.77	04.94	04.87	04.29	04.86	04.60	03.26	04.11	04.58	05.18	05.48	08.47	06.24	12.83	11.57	04.05	06.05	**0.00**

NC= Not calculated
