# Supplementary figures and images for: Molecular Evidence Reveals Taxonomic Uncertainties and Cryptic Diversity in the Neotropical Catfish of the Genus Pimelodus (Siluriformes: Pimelodidae)
Source: Biology (Basel). 2024 Mar 2;13(3):162. doi: 10.3390/biology13030162 (PMC10968110; doi:10.3390/biology13030162)

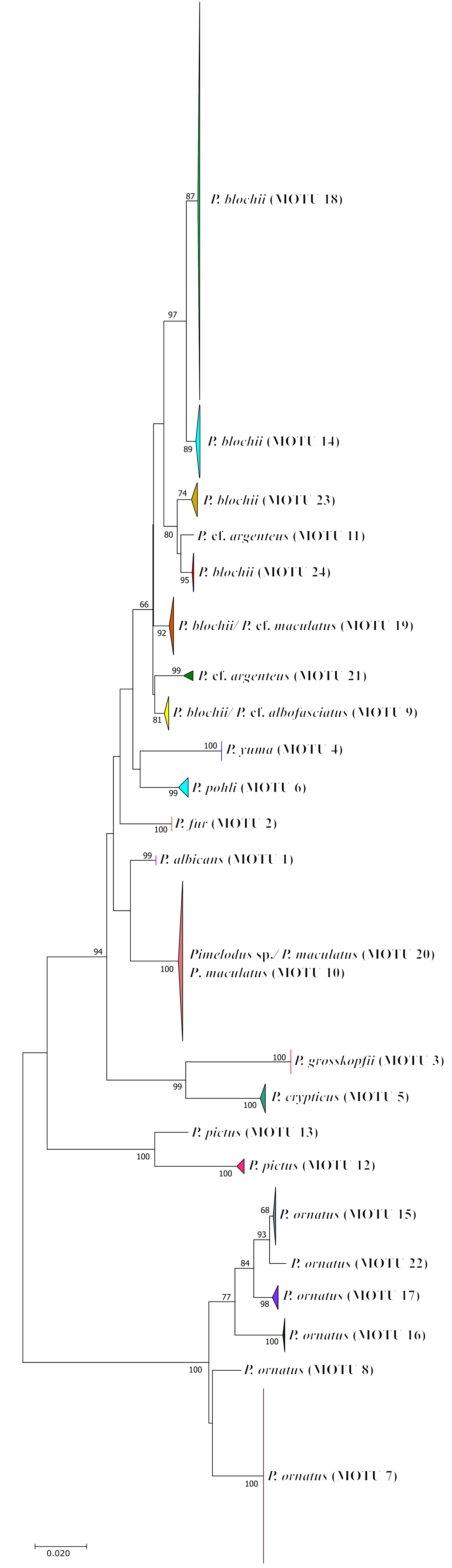

Supplement: Supplementary file 1 [file biology-13-00162-s001.zip › Figura S1 ╡rvore de Neighbour-Joining (NJ)..jpg]
